# History of trastuzumab: a case study in health technology reassessment and natural disinvestment in Veneto Region

**DOI:** 10.3389/fphar.2024.1406351

**Published:** 2024-08-06

**Authors:** Antonella Giorgia Becchetti, Anna Martini, Giovanna Scroccaro, Roberta Joppi

**Affiliations:** ^1^ Territorial Pharmaceutical Assistance Unit, Azienda ULSS 9 Scaligera, Verona, Italy; ^2^ Department Diagnostic and Public Health, University of Verona, Verona, Italy; ^3^ Direction of Pharmaceutical–Prosthetics and Medical Devices, Venice, Italy

**Keywords:** disinvestment, health technology reassessment (HTR), health technology assessment (HTA), pharmaceutical governance, trastuzumab, pharmacoeconomics, real-world data, biosimilar adoption and commercialization

## 1 Introduction

Breast cancer is the most prevalent malignancy among women worldwide, with an age-standardized rate of 46.8 per 100,000 and represents an important health burden (WHO International Agency for Research on Cancer, 2022).

In Italy, the incidence of breast cancer has been steadily increasing, with approximately 55,900 new cases diagnosed in 2022 (Lei et al., 2021; AIRTUM, 2023).

HER2-positive breast cancer, characterized by overexpression of the human epidermal growth factor receptor 2, constitutes about 20%–25% of all breast cancer cases and is associated with a more aggressive disease course and poorer prognosis (Slamon et al., 1989; Harbeck et al., 2019).

The treatment landscape for breast cancer (von Arx et al., 2023), particularly concerning biologically originated oncological medications, has witnessed significant evolution in recent years. Among these medications, trastuzumab stands as a cornerstone in the management of HER2-positive breast cancer, improving patient outcomes.

Anti-HER2 agents target HER2 receptors, inhibiting signal transduction pathways and thereby reducing cellular proliferation in HER2-positive breast neoplasms. Currently, utilized agents include trastuzumab, lapatinib, pertuzumab, trastuzumab emtansine (T-DM1), and trastuzumab deruxtecan (T-Dxd). Additionally, neratinib, a recently introduced anti-HER2 drug, may also be employed for the treatment of HER2-positive disease.

In the systemic management of metastatic breast cancer, trastuzumab may be administered in conjunction with pertuzumab and chemotherapy or combined with lapatinib.

In subsequent treatment lines, trastuzumab can be formulated as an antibody-drug conjugate (ADC), such as trastuzumab emtansine or trastuzumab deruxtecan.

Considering adjuvant therapy, trastuzumab can be utilized alongside chemotherapy, particularly taxanes, with or without endocrine therapy, if the carcinoma is hormone-responsive.

The results obtained in patients with advanced disease have provided the rationale for testing the same treatments in earlier stages, where perioperative pharmacological treatments aim to increase the chances of cure. A historical example is the result achieved with the anti-HER2 monoclonal antibody trastuzumab, which, after proving activity in combination with chemotherapy in women with HER2-positive metastatic breast cancer, has also become the standard adjuvant treatment for surgically treated patients with this molecular alteration since 2005 (AIRTUM, 2023). Alongside therapeutic advancements, economic considerations on the utilization of these medications have become increasingly pertinent (Dai et al., 2021).

High prevalence of breast carcinoma and the multifaceted spectrum of treatment modalities, coupled with the distinctive profile of trastuzumab as the inaugural HER-2 targeted therapy integrated into the therapeutic armamentarium for breast carcinoma management, encompassing both early-stage and metastatic scenarios, has steered the investigative spotlight onto this seminal agent (Maadi et al., 2021).

Amidst the myriad conjugated or combinatory formulations pervading the clinical milieu, trastuzumab ADCs are instigating paradigm shifts in the diverse demographic cohorts they cater to. This delineation underscores the pivotal role of trastuzumab and its derivative formulations in reshaping the landscape of breast cancer therapeutics, heralding a new era of precision medicine tailored to the individualised needs of patients.

### 1.1 Mechanism of action of trastuzumab

Trastuzumab was developed from the 4D5 antibody candidate and is a recombinant humanised IgG1 monoclonal antibody (Carter et al., 1992). This first-in-class drug received FDA approval in 1998. Trastuzumab specifically targets the human epidermal growth factor receptor 2 (HER2), which is overexpressed in about 20%–30% of primary breast cancers (Slamon et al., 2001). This overexpression is linked to more aggressive tumour growth, poorer prognosis, and shorter disease-free survival compared to tumours that do not overexpress HER2. Trastuzumab mechanism of action involves both direct effects on cancer cells and indirect effects on the tumour microenvironment (Spector and Blackwell, 2009).

Trastuzumab binds with high affinity and specificity to sub-domain IV, a juxta-membrane region of HER2 extracellular domain. (Baselga and Albanell, 2001; Molina et al., 2001; Baselga et al., 2001; Kreutzfeldt et al., 2020). This binding inhibits HER2 signalling that does not require a ligand (Hudis, 2007; Valabrega et al., 2007) and prevents the proteolytic cleavage of HER2’s extracellular domain, which is a key activation mechanism for HER2 (Albanell et al., 2003). Consequently, Trastuzumab has been shown to inhibit the proliferation of HER2-overexpressing human tumour cells both *in vitro* assays and animal models (Kroemer et al., 2015). Additionally, Trastuzumab effectively mediates antibody-dependent cell-mediated cytotoxicity (ADCC) (Hudis, 2007; Karamouzis et al., 2007; Greenblatt and Khaddour, 2024). *In vitro*, Trastuzumab-mediated ADCC preferentially targets cancer cells that overexpress HER2 compared to those that do not (Hudis, 2007).

### 1.2 Antibody-drug conjugates of trastuzumab

Antibody-drug conjugates (ADCs) represent a novel class of targeted cancer therapies, proving promising efficacy with tolerable systemic toxicity compared to standalone cytotoxic drugs (Patel et al., 2020). ADCs consist of a monoclonal antibody (mAb) conjugated to a cytotoxic agent (payload) via a chemical linker.

Specific antibodies targeting surface proteins of cancer cells, such as trastuzumab, offer tumour specificity, and potency unattainable with traditional drugs. The design of effective antibody-drug conjugates for cancer therapy necessitates the selection of an appropriate target, such as HER-2 (Najjar et al., 2022; Tarantino et al., 2022), a monoclonal antibody against the target, potent cytotoxic effector molecules and the linker of the monoclonal antibody to cytotoxic agents (Thomas et al., 2016).

Trastuzumab emtansine (T-DM1), the first ADC to receive Food and Drug Administration (FDA) approval for breast cancer in 2013, has proven effective in HER2+ metastatic breast cancer (Beck et al., 2017). It has significantly altered the treatment paradigm and has now become a standard second-line option for patients with this tumour type (Patel et al., 2020).

ADCs are rapidly advancing in the field of breast cancer treatment. The unique structure of ADCs, comprising a monoclonal antibody linked to a cytotoxic agent through a chemical linker, allows for specific cytotoxicity against cancer cells (Patel et al., 2020). The approval of trastuzumab emtansine has spurred further research, additional ADC candidates currently under clinical evaluation (Patel et al., 2020; von Arx et al., 2023; Swain et al., 2023). This progress highlights the significant potential of ADCs in improving cancer therapy by combining the specificity of monoclonal antibodies with the potent cytotoxic effects of traditional chemotherapy (Liang et al., 2024). Trastuzumab emtansine (T-DM1).

Trastuzumab emtansine (T-DM1) is an antibody-drug conjugate comprising trastuzumab linked to the cytotoxic agent emtansine (DM1), a maytansine derivative and microtubule inhibitor (LoRusso et al., 2011). The conjugation of DM1 to trastuzumab enhances the selectivity of the cytotoxic agent for HER2-overexpressing tumour cells, thereby improving intracellular delivery of DM1 directly to malignant cells. Upon binding to HER2, trastuzumab emtansine undergoes receptor-mediated internalisation and subsequent lysosomal degradation, resulting in the release of DM1-containing cytotoxic catabolites, primarily lysine-MCC-DM1 (Barok et al., 2014).

The DM1 component of T-DM1 binds to tubulin. By inhibiting tubulin polymerisation, DM1 and trastuzumab emtansine induce cell cycle arrest in the G2/M phase, ultimately leading to apoptotic cell death. *In vitro* cytotoxicity assays indicate that DM1 is 20–200 times more potent than taxanes and vinca alkaloids (Lambert and Chari, 2014).

The MCC linker is designed to minimise systemic release and increase targeted delivery of DM1, as demonstrated by the detection of very low levels of free DM1 in plasma (von Minckwitz et al., 2019). This targeted approach enhances the therapeutic index of T-DM1, making it as a potent treatment for HER2-positive cancers.

Trastuzumab deruxtecan (T-DXd) is a HER2-targeted antibody-drug conjugate (ADC) composed of a humanised anti-HER2 IgG1, trastuzumab linked to deruxtecan, a topoisomerase I inhibitor (DXd), through a tetrapeptide-based cleavable linker (Najjar et al., 2022). This ADC remains stable in plasma and functions by binding to HER2 expressed on certain tumour cells (Kou et al., 2023). Following binding, the trastuzumab deruxtecan complex undergoes internalisation and intracellular linker cleavage by lysosomal enzymes, which is upregulated in cancer cells. The released DXd, a membrane-permeable exatecan derivative, induces DNA damage and apoptotic cell death, exhibiting approximately 10 times higher potency than SN-38, the active metabolite of irinotecan (Xu et al., 2019; Bartsch R., 2020).

*In vitro* studies demonstrate that the antibody portion of trastuzumab deruxtecan, sharing the same amino acid sequence as trastuzumab, also binds to FcγRIIIa and complement C1q, mediating antibody-dependent cellular cytotoxicity (ADCC) in human breast cancer cells overexpressing HER2 (Hudis, 2007; Greenblatt and Khaddour, 2022). Furthermore, the antibody inhibits signalling through the phosphatidylinositol 3-kinase (PI3-K) pathway in human breast cancer cells overexpressing HER2 (Yang et al., 2023).

#### 1.2.1 Advantages of antibody-drug conjugates (ADCs)

It has been observed that ADCs with non-cleavable linkers release membrane-impermeable cytotoxic complexes and exhibit a reduced bystander effect compared to those with cleavable linkers (Erickson et al., 2010). The bystander effect refers to the phenomenon where cytotoxic payloads leak from the cell or are released extra-cellularly, killing neighbouring cells, including tumour cells that do not express the antigen (Staudacher and Brown, 2017). This effect can be leveraged to enhance therapeutic efficacy in heterogeneous or low antigen-expressing tumours (Makawita and Meric-Bernstam, 2020).

Beyond the intracellular release of the cytotoxic payload, ADCs can induce antitumor immune activity. For instance, T-DM1 has been shown to increase the number of tumour-infiltrating lymphocytes in human primary breast tumours and to induce effector T cell infiltration in murine breast tumours. In murine models, combining T-DM1 with blockade of the inhibitory PD-1/CTLA-4 pathway significantly improved T cell responses and overcame primary resistance to immune checkpoint-blocking antibodies (Müller et al., 2015).

Generally, these ADCs are well-tolerated, with toxic effects consistent with the known mechanism of action of the cytotoxic payloads, such as elevated hepatic aminotransferase levels with T-DM1. The exact mechanisms of the toxic effects attributable to ADCs are complex, involving contributions from each component of the conjugate: the monoclonal antibody, the linker, and the cytotoxic payload. Mechanisms include the nonspecific systemic release of the cytotoxic drug due to premature linker cleavage and the internalization of the ADC by cells that do not express the target. For instance, thrombocytopenia, a common toxic effect of T-DM1, is believed to result from the internalization of the ADC by megakaryocyte precursors through an Fc receptor-mediated process, after which the DM1 cytotoxic component of T-DM1 prevents differentiation into mature megakaryocytes and subsequent platelet formation (Thomas et al., 2016).

#### 1.2.2 Disadvantages of antibody-drug conjugates (ADCs)

Linkers are essential for attaching the cytotoxic payload to monoclonal antibodies (mAbs) and maintaining the structural integrity of ADCs. Their stability in the bloodstream and ability to cleave within target cells are crucial for preventing systemic toxicity from premature cleavage. The chemistry of the linker significantly affects ADC stability and the amount of payload released in the plasma, making it a primary factor in ADC toxicity (Saber and Leighton, 2015; Lu et al., 2016; Ma et al., 2023).

Compared to cleavable linkers, non-cleavable linkers provide superior plasma stability and a longer half-life, leading to reduced systemic toxicity (Peters and Brown, 2015; Ma et al., 2023). However, the choice of linker also influences the therapeutic efficacy and safety profile of ADCs. While non-cleavable linkers reduce systemic side effects, they may limit the release of the cytotoxic payload within the tumour environment, potentially affecting the overall effectiveness of the treatment.

The design of the linker must balance stability and release characteristics to maximize therapeutic benefits while minimizing adverse effects. Premature cleavage of the linker can lead to the release of the cytotoxic drug in non-target tissues, causing off-target toxicity. This is particularly concerning for cleavable linkers, which can be more susceptible to enzymatic degradation in the bloodstream (Morgovan et al., 2024).

Moreover, the internalization and processing of ADCs by cells that do not express the target antigen can also contribute to toxicity. For instance, thrombocytopenia, a common adverse effect of trastuzumab emtansine, is believed to result from the internalization of the ADC by megakaryocyte precursors through an Fc receptor-mediated process. This internalization prevents the differentiation of these precursors into mature megakaryocytes, thereby reducing platelet formation (Thomas et al., 2016).

Future advancements in ADC technology will likely focus on optimizing linker chemistry and improving target specificity to enhance safety and efficacy (Thomas et al., 2016).

#### 1.2.3 Resistance to ADCs

Due to the complexity of ADC structures and their multi-step mechanisms of action, resistance can occur at various stages, starting from antigen expression and recognition (Loganzo et al., 2015), through internalization (Scaltriti et al., 2007) and degradation, to the release of cytotoxic drugs and apoptotic regulation (Phillips et al., 2014; Chen et al., 2023; Khoury et al., 2023).

A variety of new strategies have been developed to improve ADC efficacy and overcome resistance. These include optimizing linker chemistry and conjugation methods, as well as antibody engineering. Additionally, combining ADCs with chemotherapy or targeted therapies and with immunotherapy has shown promise (Ocaña et al., 2020; Loi et al., 2021; Chen et al., 2023).

### 1.3 Mechanism of action of trastuzumab and pertuzumab

Pertuzumab and trastuzumab together constitute the therapeutic core of this medication, complemented by vorhyaluronidase alfa, an enzyme enhancing dispersion and absorption of co-formulated substances when administered subcutaneously.

Both pertuzumab and trastuzumab are recombinant humanised IgG1 monoclonal antibodies designed to target the human epidermal growth factor receptor 2 (HER2). They bind to distinct subdomains of HER2 without competing and disrupt HER2 signalling via complementary mechanisms (Pal and Pegram, 2006).

Pertuzumab specifically targets the extracellular dimerization domain (subdomain II) of HER2, inhibiting ligand-dependent heterodimerization with other HER family members, including epidermal growth factor receptor (EGFR), HER3, and HER4 (Sakai et al., 2007; Yao et al., 2009). This action blocks ligand-activated intracellular signalling through the mitogen-activated protein (MAP) kinase and phosphoinositide 3-kinase (PI3K) pathways, leading to cell growth arrest and apoptosis (Hudis, 2007; Sakai et al., 2007; Valabrega et al., 2007; Yao et al., 2009).

Moreover, both pertuzumab and trastuzumab facilitate antibody-dependent cell-mediated cytotoxicity (ADCC), with preferential exertion on HER2-overexpressing cancer cells *in vitro* compared to those lacking HER2 overexpression (Hudis, 2007; Greenblatt and Khaddour, 2024).

In healthcare systems worldwide, the concept of disinvestment has gained traction as a strategic approach to optimize resource allocation and expenditure (Polisena et al., 2013; Soril et al., 2022). Disinvestment entails the reallocation or reduction of resources from interventions or medications that are deemed less effective or cost-inefficient, thereby redirecting resources towards more beneficial and cost-effective alternatives (Mitton et al., 2019).

In the context of oncological therapy, understanding the natural disinvestment in the utilization of biologically originated medications is essential for informed decision-making and sustainable healthcare delivery. (Hollingworth and Chamberlain, 2011; Parkinson et al., 2015; Chambers et al., 2017; Pace et al., 2020; Kamaruzaman et al., 2022).

## 2 Aim of the analysis

This study aims at investigating the phenomenon of disinvestment in the Veneto Region in the utilization of biologically originated oncological medications for the treatment of breast cancer, with a specific focus on *trastuzumab*. Through a comprehensive epidemiological and pharmacoeconomic analysis, we seek to elucidate trends in pharmaceutical investment and utilization patterns over a five-year period. By examining the utilization of trastuzumab across different formulations and administration routes, we aim to provide insights crucial for informed decision-making in healthcare resource allocation and policy formulation.

We analysed trastuzumab utilization due to its status as a seminal case in the treatment of HER-2 positive breast cancer. Trastuzumab is one of the first monoclonal antibodies used on a large scale, available in different formulations (intravenous, subcutaneous, and biosimilars), as well as in antibody-drug conjugated versions and in combination with other monoclonal antibodies.

This variety of formulations allows the examination of a wide range of use and expenditure dynamics, providing a comprehensive view of the economic and clinical implications of its adoption.

The example of trastuzumab enables to illustrate how the Health Technology Assessment (HTA) approach, including the disinvestment step, can optimize resource reallocation enhancing the efficiency of the public healthcare system.

## 3 Materials and methods

### 3.1 Data source

In this descriptive study, data analysis was conducted utilizing the DataWareHouse (DWH) of the Veneto Region, which serves as a source of anonymized administrative data pertaining to consumption, expenditure, and users of medications provided through the regional healthcare service (RHS). Specifically, administrative flows from the hospital setting (FAROSP and DDF3 Canale A) were utilized.

The utilization of the DWH allowed for a comprehensive examination of medication usage trends and associated economic factors within the Veneto Region, providing valuable insights into the dynamics of healthcare resource allocation and pharmaceutical expenditure, and offering a robust foundation for evidence-based decision-making and policy development in healthcare management.

These records have been incorporated into the data analysis spanning from January 2019 to December 2023. The dataset encompasses all essential fundamental details required for the analysis, including classification for reimbursement purposes and monthly quantities of packages dispensed to each patient.

### 3.2 Estimation of medicines use

For our analysis all formulations containing trastuzumab by utilizing the Anatomical Therapeutic Chemical (ATC) classification system (WHO – https://atcddd.fhi.no/atc_ddd_index/) were identified. We included formulations such as L01FD01 *trastuzumab* intravenous (IV) and subcutaneous (SC), formulations-drug conjugated including L01FD03 *trastuzumab emtansine*, L01FD04 *trastuzumab deruxtecan* and the combination therapy L01FY01 *pertuzumab-trastuzumab*.

The ATC L01FD05 *trastuzumab duocarmazine* was excluded from the analysis as it was not utilized during the study period.

The utilization of the different formulations was analysed based on the total number of dispensed packages and the number of packages distributed per 100,000 inhabitants.

Pharmacoeconomic analyses were conducted verifying the expenditure for each formulation in every year, calculated based on the purchasing costs for the RHS. Additionally, an analysis regarding the average expenditure per patient was carried out.

A sub-analysis was conducted stratifying different formulations of trastuzumab based on the unique Italian Marketing Code for each medicine (MINSAN) and distinguishing between originator (IV or SC) and biosimilar (IV) formulations. The same analytical method described for the general analysis was applied to this stratification.

### 3.3 Indicators

To conduct an analysis with minimal bias, pharmacoeconomic data weighted by the inhabitants of the Veneto Region or weighted by treated patients were utilized. Specifically, the consumption of a specific medication was weighted per 100,000 inhabitants.

The expenditure distribution was calculated using the following indicators:

*Net per capita expenditure*: this indicator represents the net expenditure per person within the regional population. It is used for budget planning and allocation, as well as for comparing different areas or time periods. It is calculated by dividing the total net expenditure (the sum of all expenditure generated by prescription, provision, and administration of trastuzumab in its various formulations and medicinal specialties) by the number of inhabitants of the Veneto Region for each year of interest.

*Net expenditure per patient*: this indicator is calculated by dividing the global net expenditure by the total number of patients receiving at least one administration of trastuzumab during the analysis period. This indicator helps to compare the expenditure across different populations with breast cancer based on their treatments.

Additionally, data on consumption, treated patients or expenditure were utilized to compare different formulations of trastuzumab.

Population data were sourced from the figures published annually on January 1 by the Italian National Institute of Statistics (ISTAT), considering the population residing in Veneto Region (*IstatData* – The database of ISTAT https://esploradati.istat.it/databrowser/). Office suite tools such as Microsoft Excel^®^, Microsoft Access^®^, and Power BI^®^ were used.

### 3.4 Ethical considerations

This study did not require ethical review as it involved aggregated and anonymized data.

## 4 Results

Patient data, receiving at least one treatment with trastuzumab, were extracted from administrative flows, covering the period from January 2019 to December 2023.

### 4.1 Utilization data

A total of 4,321 patients received trastuzumab-based medications (ATC: L01FD01, L01FD03, L01FD04, L01FY01) (Supplementary Figure S1). An increase from 15 patients per 100,000 inhabitants in January 2019 to 21 patients per 100,000 inhabitants in December 2023 (Supplementary Table S1) was recorded, resulting in a monthly utilization rate of 19 patients per 100,000 inhabitants (Supplementary Table S1). Over the five-year analysis period, a total of 147,237 medication packages were dispensed, with a cumulative expenditure exceeding €50 million.

The number of dispensed packages increased from October 2019 to February 2020, followed by a stabilization until July 2022 and a steadily decrease until December 2023. A pick of 73 packages per 100,000 inhabitants in January 2020 vs. a nadir of 35 packages per 100,000 inhabitants in April 2023 were observed (Supplementary Tables S2, S3).

**FIGURE 1:**
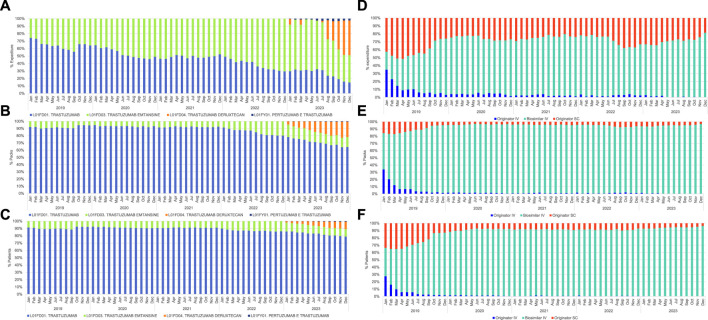
Monthly (%) percentage of trastuzumab across different domains, covering the period from January 2019 to December 2023. **(A)** Net NHS expenditure each ATC code: L01FD01 trastuzumab, L01FD03 trastuzumab emtansine, L01FD04 trastuzumab deruxtecan, L01FY01 pertuzumab-trastuzumab; **(B)** Number of dispensed packages each ATC code: L01FD01 trastuzumab, L01FD03 trastuzumab emtansine, L01FD04 trastuzumab deruxtecan, L01FY01 pertuzumab-trastuzumab; **(C)** Number of patients treated with formulations of trastuzumab under ATC codes: L01FD01 trastuzumab, L01FD03 trastuzumab emtansine, L01FD04 trastuzumab deruxtecan, L01FY01 pertuzumab-trastuzumab; **(D)** Net NHS expenditure for different formulations of trastuzumab (ATC code L01FD01): originator IV, biosimilar IV, and originator SC; **(E)** Number of dispensed packages for different formulations of trastuzumab (ATC code L01FD01): originator IV, biosimilar IV, and originator SC; **(F)** Number of patients treated with different formulations of trastuzumab (ATC code L01FD01) originator IV, biosimilar IV, and originator SC.

At regional level, from January 2019 until February 2022, the consumption of the active principle *trastuzumab* covered over 90% of all the formulations containing *trastuzumab*. The use of this drug declined gradually reaching 64% of the whole formulations in December 2023. (Figure 1B; Supplementary Table S7).

In November and December 2022 *trastuzumab emtansine* reaches 20% of all the dispensed packages corresponding to 14% of the treated patients. (Figures 1B, C; Supplementary Tables S7, S8).

In January 2023, with the introduction onto the market of *trastuzumab deruxtecan*, the utilization of *trastuzumab emtansine* decreased steadily reaching 14% of the whole formulations, while *trastuzumab deruxtecan* stabilized at 21% of the dispensed packages and 7% of the treated patients in the final quarter of 2023. (Figures 1B, C; Supplementary Tables S7, S8).

The utilization of the pre-formulated *pertuzumab-trastuzumab* combination was around 1% in terms of dispensed packages and treated subjects. (Figures 1B, C; Supplementary Tables S7, S8).

### 4.2 Sub-analysis of trastuzumab: originator IV, biosimilar IV

Over 80% of patients undergoing treatment with *trastuzumab* utilize the non-conjugated formulation (ATC: L01FD01) (Supplementary Table S8). In 2018 biosimilar of IV *trastuzumab* (ATC: L01FD01) reached the market at EU level (Harris et al., 2017). Its penetration into the regional market started in January 2019 with a dramatic decrease in terms of expenditure, dispensed packages and percentage of patients receiving the IV originator since the second half of 2019. (Figures 1D–F; Supplementary Tables S9–S11).

### 4.3 Expenditure trend

Net expenditure per inhabitant and net expenditure per patient have been analysed. A decrease in *per capita* expenditure in the first quarter of 2019 (max. €0.31 per inhabitant in January 2019 versus € 0.13 per inhabitant in July 2023), with the highest annual *per capita* expenditure recorded in 2021 at € 1.98 per inhabitant (Supplementary Table S4) has been detected.

Net expenditure per patient shows a continuous decrease from 2019 (annual average: € 1,483 *per patient*) to 2023 (annual average: € 788 *per patient*) (Supplementary Table S5).

In Figure 1, the expenditure of different trastuzumab formulations, (A), consumption (B), and utilization pattern (C) are depicted. Until December 2022, the available formulations were *trastuzumab* and *trastuzumab emtansine*. In January 2023, healthcare facilities in the Veneto Region began to utilize *trastuzumab deruxtecan* and pre-formulated combination *pertuzumab-trastuzumab*.

The analysis indicates that the expenditure for *non-conjugated trastuzumab*, has decreased from 51% in June 2020 to 15% in December 2023. This trend is directly proportional to the increase of *trastuzumab emtansine* expenditure.

Over 2023 the utilization of *trastuzumab deruxtecan* leads to a decrease in the percentage of expenditure for the following active principles: *trastuzumab* (January 2023: 30% versus December 2023: 15%) and *trastuzumab emtansine* (January 2023: 63% versus December 2023: 37%). In December 2023, 47% of the total expenditure pertains to the new formulation *trastuzumab deruxtecan*.

Finally, the utilization of the pre-formulated combination *pertuzumab-trastuzumab* averages around 2% of monthly expenditure in 2023. (Figure 1A; Supplementary Table S6).

## 5 Discussion

**FIGURE 2:**
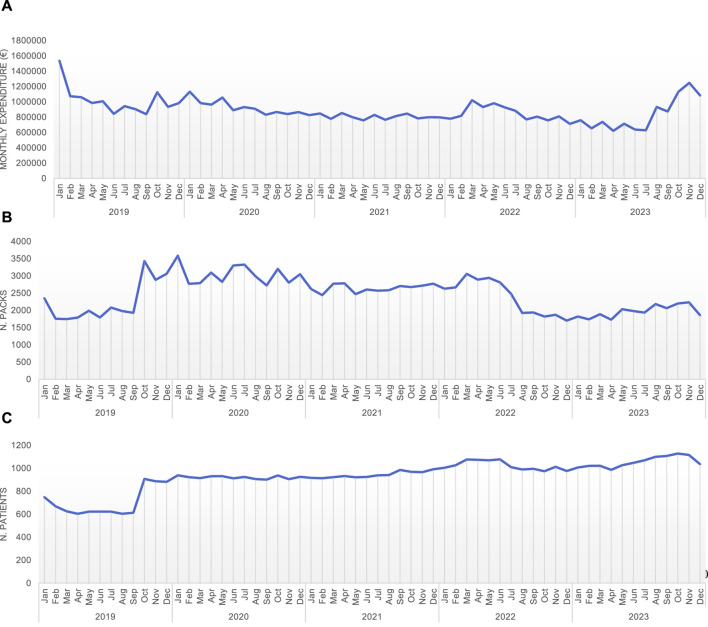
Monthly expenditure **(A)**, number of packages **(B)**, and number of patients treated **(C)** with different *trastuzumab* types or combination (*trastuzumab* – L01FD01; *trastuzumab emtansine* – L01FD03; *trastuzumab deruxtecan* – L01FD04; *pertuzumab-trastuzumab* – L01FY01).

Trastuzumab stands as a pioneering monoclonal antibody in the therapeutic arsenal against HER-2 positive breast carcinomas, notorious for their aggressive nature. The increasingly established use recommended by international guidelines has led to a more widespread utilization of this drug (Figure 2).

Since its commercial inception, diverse formulations have emerged, notably the subcutaneous variant, followed by its conjugation with other cytotoxic agents aimed at amplifying the therapeutic benefits of the drug, while mitigating its drawbacks.

Our analysis sheds light on the dynamic nature of drug utilization in clinical practice. The emergence of new formulations (*trastuzumab emtansine* and *trastuzumab deruxtecan*) played a significant role in reshaping how trastuzumab was being used. It is interesting to see how the new options gained traction, gradually overshadowing the original *trastuzumab* formulations in terms of prescription patterns.

The initial introduction of biosimilar formulations has resulted in a steady increase in their usage, while the use of the originator experienced a sharp decline in 2019, stabilizing in the subsequent years.

One of the most striking findings of our analysis was the decrease in the overall expenditure associated with *trastuzumab* utilization. This reduction was directly linked to changes in prescription patterns and the availability of more cost-effective alternatives. Initially, *trastuzumab* and *trastuzumab emtansine* dominated the market. From January 2023, the introduction of *trastuzumab deruxtecan* significantly altered the landscape, accounting for 47% of the expenditure by December 2023.

The utilization of the *pertuzumab-trastuzumab* combination remained minimal, averaging around 2% of the monthly expenditure.

These findings have important implications on how to manage healthcare resources, especially in the context of treating HER2-positive breast cancer.

An HTA approach, consumption data analysis to predict expenditure and a centralized procurement strategy contribute to a decrease in the average expenditure per patient, to expand the population receiving treatment (Figure 2) and allows to make more informed decisions about resource allocation.

### 5.1 New perspective on ADC in HER2-low and HER2-ultralow

In Italy, in 2023, there were 55,900 new cases of breast cancer, with the most common subtype being hormone receptor-positive (HR+) and HER2-negative, accounting for 70% of the total. It is estimated that approximately 60%–65% of HR-positive, HER2-negative breast cancers are actually HER2-low, with a possible additional 25% of HER2-ultralow. The use of trastuzumab deruxtecan (T-Dxd), based on literature results, presents a valuable option to expand treatment horizons for patients previously excluded from the benefits of HER2-targeted therapies (Bardia and Viale, 2023; Franchina et al., 2023).

In patients with low HER2 protein expression (HER2-low), T-Dxd has demonstrated a 38% reduction in the risk of disease progression or death, with a median progression-free survival (PFS) of 13.2 months compared to 8.1 months with standard chemotherapy.

The objective response rate also improved, reaching 56.5% compared to 32.3%. In patients with very low HER2 protein expression (HER2-ultralow) the response rate has more than doubled vs. chemotherapy (61.8% versus 26.3%) (Corti et al., 2023; Cortés et al., 2024).

The promising results of T-Dxd in HER2-low and HER2-ultralow breast cancer patients highlight a new horizon for ADCs. The potential of these conjugates to deliver targeted cytotoxicity while sparing normal tissues provides a compelling rationale for their broader application. Future research should focus on optimizing ADC chemistry, including linker and conjugation technology, and exploring combinations with other therapies such as chemotherapy and immunotherapy to overcome resistance and enhance efficacy (Ocaña et al., 2020; Loi et al., 2021; Chen et al., 2023).

## 6 Limitations of the study

This study has limitations regarding the expenditure analysis, as medications provided through the regional healthcare system are subject to negotiations that may result in expenditure variations due to an increase in the number of dispensed packages. This could introduce bias in the analysis when compared across different time periods.

Additionally, access to innovative drugs may occur through compassionate use, or the utilization of non-negotiated class C drugs (C (nn)), leading to significant variations in the actual expenditure for drugs between the pre- and post- price-negotiations at national level and the following purchase through regional tender.

Patient data were anonymous, which has prevented to verify possible therapeutic switches or continuations with subsequent lines of treatment.

## 7 Conclusion

In Italy, in 2022, an estimated 15,500 deaths occurred for breast cancer. The net five-year survival rate from diagnosis is 88% and probability of living an additional 4 years is 91%, depending on surviving the first year after diagnosis. Additionally, there are 834,200 women living in Italy after a diagnosis of breast cancer (AIRTUM, 2023).

Future developments in the field of ADCs will likely focus on identifying better targets, using novel and effective cytotoxic payloads, and further improving linker technologies to deliver more potent and safer drugs (Morgovan et al., 2024; Thomas et al., 2016).

Our analysis is part of disinvestment approach, enabling the examination of shifts in resource allocation, from less efficient or out-of-date therapies towards more cost-effective alternatives (Specchia et al., 2018). A multidisciplinary approach allowed for a thorough assessment of medication utilization patterns, expenditure trends, and cost-effectiveness considerations within the context of *trastuzumab* usage for breast cancer treatment.

By examining both the percentage of drug usage and the associated expenditure, this study provides valuable insights into the economic implications of drug utilization, aiding in resource allocation and decision-making processes within the healthcare systems (Girardin et al., 2023).

## Data Availability

The original contributions presented in the study are included in the article/Supplementary Material, further inquiries can be directed to the corresponding author.
