# Supplementary material for: History of trastuzumab: a case study in health technology reassessment and natural disinvestment in Veneto Region
Source: Front Pharmacol. 2024 Aug 6;15:1406351. doi: 10.3389/fphar.2024.1406351 (PMC11333330; doi:10.3389/fphar.2024.1406351)
Supplement: Supplementary file 1 [file Table1.DOCX]

Supplementary Material

# Supplementary Data (Update 2024)

Table S 1 – Prevalence of trastuzumab use per 100,000 inhabitants (all ATC codes)

|  | **2019** | **2020** | **2021** | **2022** | **2023** |
| --- | --- | --- | --- | --- | --- |
| **Jan** | 15.273 | 19.184 | 18.789 | 20.690 | 20.620 |
| **Feb** | 13.635 | 18.815 | 18.707 | 21.144 | 21.033 |
| **Mar** | 12.754 | 18.548 | 18.912 | 22.134 | 20.930 |
| **Apr** | 12.304 | 19.040 | 19.077 | 22.113 | 20.311 |
| **May** | 12.652 | 18.938 | 18.871 | 22.031 | 21.157 |
| **Jun** | 12.652 | 18.610 | 18.933 | 22.196 | 21.548 |
| **Jul** | 12.734 | 18.917 | 19.200 | 20.752 | 21.981 |
| **Aug** | 12.304 | 18.425 | 19.261 | 20.381 | 22.621 |
| **Sep** | 12.529 | 18.364 | 20.124 | 20.504 | 22.786 |
| **Oct** | 18.569 | 19.102 | 19.877 | 20.030 | 23.260 |
| **Nov** | 18.118 | 18.446 | 19.775 | 20.834 | 22.971 |
| **Dec** | 17.954 | 18.856 | 20.309 | 20.112 | 21.342 |

Five-year average of monthly prevalence of trastuzumab use per 100,000 inhabitants = 19,034

Table S 2 – Total number of trastuzumab packages per 100,000 inhabitants (all ATC codes)

|  | **2019** | **2020** | **2021** | **2022** | **2023** |
| --- | --- | --- | --- | --- | --- |
| **Jan** | 48.005 | 73.414 | 53.728 | 54.148 | 37.496 |
| **Feb** | 35.897 | 56.712 | 50.083 | 54.920 | 35.798 |
| **Mar** | 35.706 | 57.169 | 56.935 | 63.050 | 38.853 |
| **Apr** | 36.617 | 63.390 | 57.148 | 59.622 | 35.641 |
| **May** | 40.693 | 57.885 | 50.647 | 60.691 | 41.875 |
| **Jun** | 36.644 | 67.648 | 53.475 | 57.961 | 40.762 |
| **Jul** | 42.528 | 68.157 | 52.781 | 51.133 | 39.874 |
| **Aug** | 40.447 | 61.141 | 53.029 | 39.640 | 44.973 |
| **Sep** | 39.408 | 55.754 | 55.492 | 39.955 | 42.534 |
| **Oct** | 70.202 | 65.627 | 54.932 | 37.523 | 45.294 |
| **Nov** | 59.022 | 57.423 | 55.721 | 38.576 | 46.009 |
| **Dec** | 62.628 | 62.449 | 56.898 | 35.032 | 38.293 |
| **Total** | 547.795 | 746.7685 | 650.8697 | 592.2506 | 487.4027 |

Table S 3 – Total number of trastuzumab packages per patient (all ATC codes)

|  | **2019** | **2020** | **2021** | **2022** | **2023** |
| --- | --- | --- | --- | --- | --- |
| **Jan** | 3.143 | 3.827 | 2.859 | 2.617 | 1.818 |
| **Feb** | 2.633 | 3.014 | 2.677 | 2.597 | 1.702 |
| **Mar** | 2.800 | 3.082 | 3.010 | 2.849 | 1.856 |
| **Apr** | 2.976 | 3.329 | 2.996 | 2.696 | 1.755 |
| **May** | 3.216 | 3.057 | 2.684 | 2.755 | 1.979 |
| **Jun** | 2.896 | 3.635 | 2.824 | 2.611 | 1.892 |
| **Jul** | 3.340 | 3.603 | 2.749 | 2.464 | 1.814 |
| **Aug** | 3.287 | 3.318 | 2.753 | 1.945 | 1.988 |
| **Sep** | 3.145 | 3.036 | 2.758 | 1.949 | 1.867 |
| **Oct** | 3.781 | 3.436 | 2.764 | 1.873 | 1.947 |
| **Nov** | 3.258 | 3.113 | 2.818 | 1.852 | 2.003 |
| **Dec** | 3.488 | 3.312 | 2.802 | 1.742 | 1.794 |

Table S 4 – Total per - capita expenditure on trastuzumab (all ATC codes)

|  | **2019 (€)** | **2020 (€)** | **2021 (€)** | **2022 (€)** | **2023 (€)** |
| --- | --- | --- | --- | --- | --- |
| **Jan** | 0.31 | 0.23 | 0.17 | 0.16 | 0.16 |
| **Feb** | 0.22 | 0.20 | 0.16 | 0.17 | 0.13 |
| **Mar** | 0.22 | 0.20 | 0.18 | 0.21 | 0.15 |
| **Apr** | 0.20 | 0.22 | 0.16 | 0.19 | 0.13 |
| **May** | 0.21 | 0.18 | 0.16 | 0.20 | 0.15 |
| **Jun** | 0.17 | 0.19 | 0.17 | 0.19 | 0.13 |
| **Jul** | 0.19 | 0.19 | 0.16 | 0.18 | 0.13 |
| **Aug** | 0.18 | 0.17 | 0.17 | 0.16 | 0.19 |
| **Sep** | 0.17 | 0.18 | 0.17 | 0.17 | 0.18 |
| **Oct** | 0.23 | 0.17 | 0.16 | 0.16 | 0.23 |
| **Nov** | 0.19 | 0.18 | 0.16 | 0.17 | 0.26 |
| **Dec** | 0.20 | 0.17 | 0.16 | 0.15 | 0.22 |
| **Total** | 2.50 | 2.27 | 1.98 | 2.10 | 2.07 |

Average per capita trastuzumab expenditure (period: 2019-2023) = €2.19

Table S 5 – Total net expenditure per patients (all ATC codes)

|  | **2019 (€)** | **2020 (€)** | **2021 (€)** | **2022 (€)** | **2023 (€)** |
| --- | --- | --- | --- | --- | --- |
| **Jan** | 2,057 | 1,209 | 925 | 776 | 760 |
| **Feb** | 1,609 | 1,070 | 854 | 797 | 642 |
| **Mar** | 1,702 | 1,064 | 927 | 951 | 727 |
| **Apr** | 1,637 | 1,137 | 859 | 869 | 630 |
| **May** | 1,629 | 962 | 825 | 918 | 695 |
| **Jun** | 1,364 | 1,026 | 899 | 862 | 610 |
| **Jul** | 1,515 | 985 | 818 | 877 | 589 |
| **Aug** | 1,503 | 923 | 868 | 779 | 850 |
| **Sep** | 1,370 | 967 | 863 | 810 | 790 |
| **Oct** | 1,239 | 901 | 809 | 779 | 1,003 |
| **Nov** | 1,056 | 962 | 828 | 801 | 1,120 |
| **Dec** | 1,120 | 897 | 806 | 730 | 1,046 |
| **Annual Medium** | 1,483 | 1,009 | 857 | 829 | 788 |

Table S 6 – Net NHS expenditure of different formulations / comnbination of trastuzumab

|  | | **L01FD01** *Trastuzumab* | **L01FD03**  *Trastuzumab Emtansine* | **L01FD04**  *Trastuzumab Deruxtecan* | **L01FY01**  *Pertuzumab-Trastuzumab* |
| --- | --- | --- | --- | --- | --- |
| **2019** | *Jan* | 74% | 26% | - | - |
|  | *Feb* | 73% | 27% | - | - |
|  | *Mar* | 66% | 34% | - | - |
|  | *Apr* | 66% | 34% | - | - |
|  | *May* | 63% | 37% | - | - |
|  | *Jun* | 64% | 36% | - | - |
|  | *Jul* | 60% | 40% | - | - |
|  | *Aug* | 58% | 42% | - | - |
|  | *Sep* | 56% | 44% | - | - |
|  | *Oct* | 66% | 34% | - | - |
|  | *Nov* | 66% | 34% | - | - |
|  | *Dec* | 64% | 36% | - | - |
| **2020** | *Jan* | 65% | 35% | - | - |
|  | *Feb* | 61% | 39% | - | - |
|  | *Mar* | 62% | 38% | - | - |
|  | *Apr* | 59% | 41% | - | - |
|  | *May* | 57% | 43% | - | - |
|  | *Jun* | 51% | 49% | - | - |
|  | *Jul* | 51% | 49% | - | - |
|  | *Aug* | 49% | 51% | - | - |
|  | *Sep* | 48% | 52% | - | - |
|  | *Oct* | 47% | 53% | - | - |
|  | *Nov* | 46% | 54% | - | - |
|  | *Dec* | 49% | 51% | - | - |
| **2021** | *Jan* | 46% | 54% | - | - |
|  | *Feb* | 46% | 54% | - | - |
|  | *Mar* | 49% | 51% | - | - |
|  | *Apr* | 51% | 49% | - | - |
|  | *May* | 51% | 49% | - | - |
|  | *Jun* | 47% | 53% | - | - |
|  | *Jul* | 50% | 50% | - | - |
|  | *Aug* | 48% | 52% | - | - |
|  | *Sep* | 48% | 52% | - | - |
|  | *Oct* | 49% | 51% | - | - |
|  | *Nov* | 50% | 50% | - | - |
|  | *Dec* | 52% | 48% | - | - |
| **2022** | *Jan* | 49% | 51% | - | - |
|  | *Feb* | 46% | 54% | - | - |
|  | *Mar* | 42% | 58% | - | - |
|  | *Apr* | 44% | 56% | - | - |
|  | *May* | 42% | 58% | - | - |
|  | *Jun* | 42% | 58% | - | - |
|  | *Jul* | 36% | 64% | - | - |
|  | *Aug* | 34% | 66% | - | - |
|  | *Sep* | 32% | 68% | - | - |
|  | *Oct* | 32% | 68% | - | - |
|  | *Nov* | 30% | 70% | - | - |
|  | *Dec* | 29% | 71% | - | - |
| **2023** | *Jan* | 30% | 63% | 7% | - |
|  | *Feb* | 32% | 67% | <1% | 1% |
|  | *Mar* | 31% | 61% | 7% | 1% |
|  | *Apr* | 32% | 68% | <1% | 1% |
|  | *May* | 30% | 69% | <1% | 1% |
|  | *Jun* | 32% | 65% | <1% | 2% |
|  | *Jul* | 31% | 64% | 2% | 3% |
|  | *Aug* | 24% | 49% | 25% | 2% |
|  | *Sep* | 23% | 48% | 26% | 3% |
|  | *Oct* | 19% | 40% | 38% | 3% |
|  | *Nov* | 16% | 36% | 46% | 2% |
|  | *Dec* | 15% | 37% | 47% | 2% |

Table S 7 – Number packages of different formulations / comnbination of trastuzumab

|  | | **L01FD01** *Trastuzumab* | **L01FD03**  *Trastuzumab Emtansine* | **L01FD04**  *Trastuzumab Deruxtecan* | **L01FY01**  *Pertuzumab-Trastuzumab* |
| --- | --- | --- | --- | --- | --- |
| **2019** | *Jan* | 92% | 8% | - | - |
|  | *Feb* | 92% | 8% | - | - |
|  | *Mar* | 90% | 10% | - | - |
|  | *Apr* | 91% | 9% | - | - |
|  | *May* | 91% | 9% | - | - |
|  | *Jun* | 92% | 8% | - | - |
|  | *Jul* | 91% | 9% | - | - |
|  | *Aug* | 91% | 9% | - | - |
|  | *Sep* | 90% | 10% | - | - |
|  | *Oct* | 95% | 5% | - | - |
|  | *Nov* | 95% | 5% | - | - |
|  | *Dec* | 94% | 6% | - | - |
| **2020** | *Jan* | 95% | 5% | - | - |
|  | *Feb* | 93% | 7% | - | - |
|  | *Mar* | 94% | 6% | - | - |
|  | *Apr* | 93% | 7% | - | - |
|  | *May* | 93% | 7% | - | - |
|  | *Jun* | 93% | 7% | - | - |
|  | *Jul* | 94% | 6% | - | - |
|  | *Aug* | 93% | 7% | - | - |
|  | *Sep* | 92% | 8% | - | - |
|  | *Oct* | 93% | 7% | - | - |
|  | *Nov* | 92% | 8% | - | - |
|  | *Dec* | 93% | 7% | - | - |
| **2021** | *Jan* | 92% | 8% | - | - |
|  | *Feb* | 91% | 9% | - | - |
|  | *Mar* | 92% | 8% | - | - |
|  | *Apr* | 93% | 7% | - | - |
|  | *May* | 92% | 8% | - | - |
|  | *Jun* | 92% | 8% | - | - |
|  | *Jul* | 93% | 7% | - | - |
|  | *Aug* | 92% | 8% | - | - |
|  | *Sep* | 92% | 8% | - | - |
|  | *Oct* | 92% | 8% | - | - |
|  | *Nov* | 92% | 8% | - | - |
|  | *Dec* | 93% | 7% | - | - |
| **2022** | *Jan* | 91% | 9% | - | - |
|  | *Feb* | 90% | 10% | - | - |
|  | *Mar* | 88% | 12% | - | - |
|  | *Apr* | 88% | 12% | - | - |
|  | *May* | 87% | 13% | - | - |
|  | *Jun* | 87% | 13% | - | - |
|  | *Jul* | 85% | 15% | - | - |
|  | *Aug* | 82% | 18% | - | - |
|  | *Sep* | 81% | 19% | - | - |
|  | *Oct* | 81% | 19% | - | - |
|  | *Nov* | 80% | 20% | - | - |
|  | *Dec* | 80% | 20% | - | - |
| **2023** | *Jan* | 78% | 17% | 5% | <1% |
|  | *Feb* | 76% | 17% | 7% | <1% |
|  | *Mar* | 76% | 16% | 9% | <1% |
|  | *Apr* | 75% | 16% | 9% | <1% |
|  | *May* | 72% | 16% | 12% | <1% |
|  | *Jun* | 71% | 14% | 14% | <1% |
|  | *Jul* | 70% | 14% | 16% | <1% |
|  | *Aug* | 69% | 14% | 17% | <1% |
|  | *Sep* | 67% | 13% | 19% | <1% |
|  | *Oct* | 68% | 13% | 19% | 1% |
|  | *Nov* | 64% | 13% | 22% | <1% |
|  | *Dec* | 64% | 14% | 21% | <1% |

Table S 8 – Number of patients treated with different formulations /combination of trastuzumab

|  | | **L01FD01** *Trastuzumab* | **L01FD03**  *Trastuzumab Emtansine* | **L01FD04**  *Trastuzumab Deruxtecan* | **L01FY01**  *Pertuzumab-Trastuzumab* |
| --- | --- | --- | --- | --- | --- |
| **2019** | *Jan* | 92% | 8% | - | - |
|  | *Feb* | 91% | 9% | - | - |
|  | *Mar* | 91% | 9% | - | - |
|  | *Apr* | 89% | 11% | - | - |
|  | *May* | 89% | 11% | - | - |
|  | *Jun* | 89% | 11% | - | - |
|  | *Jul* | 90% | 10% | - | - |
|  | *Aug* | 90% | 10% | - | - |
|  | *Sep* | 89% | 11% | - | - |
|  | *Oct* | 89% | 11% | - | - |
|  | *Nov* | 92% | 8% | - | - |
|  | *Dec* | 92% | 8% | - | - |
| **2020** | *Jan* | 92% | 8% | - | - |
|  | *Feb* | 93% | 7% | - | - |
|  | *Mar* | 92% | 8% | - | - |
|  | *Apr* | 92% | 8% | - | - |
|  | *May* | 92% | 8% | - | - |
|  | *Jun* | 92% | 8% | - | - |
|  | *Jul* | 91% | 9% | - | - |
|  | *Aug* | 91% | 9% | - | - |
|  | *Sep* | 91% | 9% | - | - |
|  | *Oct* | 90% | 10% | - | - |
|  | *Nov* | 91% | 9% | - | - |
|  | *Dec* | 90% | 10% | - | - |
| **2021** | *Jan* | 90% | 10% | - | - |
|  | *Feb* | 91% | 9% | - | - |
|  | *Mar* | 90% | 10% | - | - |
|  | *Apr* | 91% | 9% | - | - |
|  | *May* | 91% | 9% | - | - |
|  | *Jun* | 91% | 9% | - | - |
|  | *Jul* | 91% | 9% | - | - |
|  | *Aug* | 91% | 9% | - | - |
|  | *Sep* | 91% | 9% | - | - |
|  | *Oct* | 91% | 9% | - | - |
|  | *Nov* | 91% | 9% | - | - |
|  | *Dec* | 91% | 9% | - | - |
| **2022** | *Jan* | 91% | 9% | - | - |
|  | *Feb* | 90% | 10% | - | - |
|  | *Mar* | 88% | 12% | - | - |
|  | *Apr* | 87% | 13% | - | - |
|  | *May* | 87% | 13% | - | - |
|  | *Jun* | 87% | 13% | - | - |
|  | *Jul* | 87% | 13% | - | - |
|  | *Aug* | 86% | 14% | - | - |
|  | *Sep* | 87% | 13% | - | - |
|  | *Oct* | 87% | 13% | - | - |
|  | *Nov* | 86% | 14% | - | - |
|  | *Dec* | 86% | 14% | - | - |
| **2023** | *Jan* | 86% | 14% | <1% | <1% |
|  | *Feb* | 86% | 12% | 2% | <1% |
|  | *Mar* | 86% | 12% | 2% | <1% |
|  | *Apr* | 85% | 12% | 3% | <1% |
|  | *May* | 85% | 12% | 4% | <1% |
|  | *Jun* | 83% | 12% | 5% | <1% |
|  | *Jul* | 83% | 11% | 6% | <1% |
|  | *Aug* | 83% | 10% | 6% | <1% |
|  | *Sep* | 82% | 11% | 7% | <1% |
|  | *Oct* | 80% | 10% | 9% | 1% |
|  | *Nov* | 80% | 10% | 9% | 1% |
|  | *Dec* | 80% | 10% | 9% | 1% |

Table S 9 – Net NHS expenditure for Trastuzumab .

| **L01FD01 Trastuzumab** | | | | |
| --- | --- | --- | --- | --- |
|  | | **Originator IV** | **Biosimilar IV** | **Originator SC** |
| **2019** | *Jan* | 35% | 23% | 42% |
|  | *Feb* | 23% | 30% | 47% |
|  | *Mar* | 15% | 34% | 51% |
|  | *Apr* | 9% | 40% | 51% |
|  | *May* | 10% | 42% | 48% |
|  | *Jun* | 10% | 43% | 47% |
|  | *Jul* | 6% | 50% | 44% |
|  | *Aug* | 5% | 50% | 45% |
|  | *Sep* | 6% | 56% | 38% |
|  | *Oct* | 4% | 68% | 28% |
|  | *Nov* | 6% | 68% | 26% |
|  | *Dec* | 3% | 71% | 26% |
| **2020** | *Jan* | 4% | 71% | 25% |
|  | *Feb* | 4% | 73% | 23% |
|  | *Mar* | 4% | 73% | 23% |
|  | *Apr* | 4% | 74% | 22% |
|  | *May* | 4% | 74% | 23% |
|  | *Jun* | 6% | 72% | 22% |
|  | *Jul* | 4% | 70% | 26% |
|  | *Aug* | 5% | 68% | 26% |
|  | *Sep* | 5% | 67% | 28% |
|  | *Oct* | 5% | 67% | 28% |
|  | *Nov* | 3% | 68% | 28% |
|  | *Dec* | 2% | 72% | 27% |
| **2021** | *Jan* | 2% | 69% | 29% |
|  | *Feb* | 3% | 71% | 26% |
|  | *Mar* | 2% | 71% | 27% |
|  | *Apr* | 3% | 72% | 25% |
|  | *May* | 4% | 71% | 25% |
|  | *Jun* | 4% | 72% | 24% |
|  | *Jul* | 2% | 77% | 21% |
|  | *Aug* | 2% | 75% | 23% |
|  | *Sep* | 2% | 74% | 24% |
|  | *Oct* | 2% | 78% | 20% |
|  | *Nov* | 2% | 74% | 23% |
|  | *Dec* | 1% | 75% | 24% |
| **2022** | *Jan* | 4% | 75% | 22% |
|  | *Feb* | 1% | 76% | 23% |
|  | *Mar* | 3% | 76% | 21% |
|  | *Apr* | 1% | 75% | 24% |
|  | *May* | 2% | 75% | 23% |
|  | *Jun* | 1% | 75% | 23% |
|  | *Jul* | 3% | 69% | 29% |
|  | *Aug* | 3% | 63% | 34% |
|  | *Sep* | 3% | 59% | 38% |
|  | *Oct* | 4% | 59% | 37% |
|  | *Nov* | 2% | 60% | 37% |
|  | *Dec* | 2% | 65% | 33% |
| **2023** | *Jan* | 3% | 64% | 33% |
|  | *Feb* | 1% | 65% | 34% |
|  | *Mar* | 2% | 64% | 34% |
|  | *Apr* | 2% | 68% | 30% |
|  | *May* | 0% | 71% | 29% |
|  | *Jun* | 0% | 71% | 28% |
|  | *Jul* | 1% | 73% | 27% |
|  | *Aug* | 1% | 70% | 29% |
|  | *Sep* | 0% | 73% | 27% |
|  | *Oct* | 1% | 72% | 28% |
|  | *Nov* | 0% | 76% | 24% |
|  | *Dec* | 0% | 81% | 19% |

Table S 10 – Number of Trastuzumab packages

| **L01FD01 Trastuzumab** | | | | |
| --- | --- | --- | --- | --- |
|  | | **Originator IV** | **Biosimilar IV** | **Originator SC** |
| **2019** | *Jan* | 34% | 50% | 16% |
|  | *Feb* | 20% | 63% | 17% |
|  | *Mar* | 12% | 71% | 17% |
|  | *Apr* | 7% | 78% | 16% |
|  | *May* | 7% | 79% | 14% |
|  | *Jun* | 7% | 81% | 13% |
|  | *Jul* | 4% | 85% | 11% |
|  | *Aug* | 3% | 86% | 11% |
|  | *Sep* | 3% | 88% | 9% |
|  | *Oct* | 2% | 93% | 5% |
|  | *Nov* | 2% | 93% | 5% |
|  | *Dec* | 1% | 94% | 5% |
| **2020** | *Jan* | 2% | 94% | 4% |
|  | *Feb* | 2% | 94% | 4% |
|  | *Mar* | 2% | 94% | 4% |
|  | *Apr* | 2% | 94% | 4% |
|  | *May* | 1% | 95% | 4% |
|  | *Jun* | 2% | 95% | 3% |
|  | *Jul* | 1% | 96% | 3% |
|  | *Aug* | 2% | 95% | 3% |
|  | *Sep* | 1% | 95% | 4% |
|  | *Oct* | 1% | 96% | 3% |
|  | *Nov* | 1% | 95% | 4% |
|  | *Dec* | 0% | 96% | 3% |
| **2021** | *Jan* | 1% | 95% | 4% |
|  | *Feb* | 1% | 96% | 4% |
|  | *Mar* | 1% | 95% | 4% |
|  | *Apr* | 1% | 95% | 4% |
|  | *May* | 1% | 94% | 4% |
|  | *Jun* | 2% | 94% | 4% |
|  | *Jul* | 1% | 95% | 4% |
|  | *Aug* | 1% | 95% | 4% |
|  | *Sep* | 1% | 94% | 4% |
|  | *Oct* | 1% | 95% | 4% |
|  | *Nov* | 1% | 95% | 4% |
|  | *Dec* | 1% | 95% | 5% |
| **2022** | *Jan* | 2% | 94% | 4% |
|  | *Feb* | 1% | 95% | 4% |
|  | *Mar* | 1% | 95% | 4% |
|  | *Apr* | 1% | 95% | 4% |
|  | *May* | 1% | 95% | 4% |
|  | *Jun* | 1% | 95% | 4% |
|  | *Jul* | 1% | 94% | 5% |
|  | *Aug* | 2% | 92% | 7% |
|  | *Sep* | 1% | 91% | 8% |
|  | *Oct* | 2% | 91% | 7% |
|  | *Nov* | 1% | 92% | 7% |
|  | *Dec* | 1% | 93% | 6% |
| **2023** | *Jan* | 1% | 92% | 6% |
|  | *Feb* | 1% | 93% | 6% |
|  | *Mar* | 1% | 93% | 7% |
|  | *Apr* | 1% | 94% | 6% |
|  | *May* | 0% | 95% | 5% |
|  | *Jun* | 0% | 95% | 5% |
|  | *Jul* | 0% | 95% | 5% |
|  | *Aug* | 0% | 95% | 5% |
|  | *Sep* | 0% | 95% | 5% |
|  | *Oct* | 0% | 95% | 5% |
|  | *Nov* | 0% | 96% | 4% |
|  | *Dec* | 0% | 97% | 3% |

Table S 11 – Number of patients treated with Trastuzumab .

| **L01FD01 Trastuzumab** | | | | |
| --- | --- | --- | --- | --- |
|  | | **Originator IV** | **Biosimilar IV** | **Originator SC** |
| **2019** | *Jan* | 27% | 39% | 34% |
|  | *Feb* | 16% | 49% | 35% |
|  | *Mar* | 10% | 55% | 35% |
|  | *Apr* | 6% | 59% | 35% |
|  | *May* | 6% | 63% | 32% |
|  | *Jun* | 6% | 65% | 29% |
|  | *Jul* | 3% | 70% | 27% |
|  | *Aug* | 2% | 72% | 26% |
|  | *Sep* | 3% | 75% | 22% |
|  | *Oct* | 2% | 85% | 13% |
|  | *Nov* | 2% | 85% | 13% |
|  | *Dec* | 1% | 86% | 13% |
| **2020** | *Jan* | 1% | 87% | 12% |
|  | *Feb* | 1% | 88% | 10% |
|  | *Mar* | 2% | 88% | 10% |
|  | *Apr* | 1% | 90% | 9% |
|  | *May* | 1% | 91% | 8% |
|  | *Jun* | 1% | 92% | 7% |
|  | *Jul* | 1% | 91% | 8% |
|  | *Aug* | 1% | 92% | 7% |
|  | *Sep* | 1% | 91% | 8% |
|  | *Oct* | 1% | 91% | 8% |
|  | *Nov* | 1% | 91% | 8% |
|  | *Dec* | 0% | 92% | 8% |
| **2021** | *Jan* | 0% | 91% | 9% |
|  | *Feb* | 0% | 91% | 8% |
|  | *Mar* | 0% | 91% | 8% |
|  | *Apr* | 1% | 91% | 8% |
|  | *May* | 1% | 90% | 8% |
|  | *Jun* | 1% | 91% | 8% |
|  | *Jul* | 1% | 91% | 8% |
|  | *Aug* | 0% | 91% | 9% |
|  | *Sep* | 1% | 91% | 8% |
|  | *Oct* | 1% | 91% | 8% |
|  | *Nov* | 1% | 91% | 9% |
|  | *Dec* | 1% | 90% | 9% |
| **2022** | *Jan* | 1% | 91% | 8% |
|  | *Feb* | 0% | 91% | 9% |
|  | *Mar* | 1% | 91% | 8% |
|  | *Apr* | 0% | 90% | 9% |
|  | *May* | 0% | 91% | 8% |
|  | *Jun* | 0% | 91% | 9% |
|  | *Jul* | 1% | 90% | 9% |
|  | *Aug* | 1% | 91% | 8% |
|  | *Sep* | 1% | 89% | 10% |
|  | *Oct* | 1% | 90% | 9% |
|  | *Nov* | 1% | 90% | 9% |
|  | *Dec* | 1% | 92% | 7% |
| **2023** | *Jan* | 0% | 92% | 7% |
|  | *Feb* | 0% | 93% | 7% |
|  | *Mar* | 0% | 93% | 7% |
|  | *Apr* | 0% | 93% | 6% |
|  | *May* | 0% | 94% | 6% |
|  | *Jun* | 0% | 94% | 6% |
|  | *Jul* | 0% | 95% | 5% |
|  | *Aug* | 0% | 94% | 5% |
|  | *Sep* | 0% | 94% | 6% |
|  | *Oct* | 0% | 94% | 5% |
|  | *Nov* | 0% | 95% | 5% |
|  | *Dec* | 0% | 96% | 4% |

# Supplementary Figure (update March 2024)

Supplementary Figure 1 – Number of patients receiving at least once Trastuzumab medication from January 2019 to December 2023 in the Veneto Region (all ATC Codes).
